# Carfilzomib-Induced Tumor Lysis Syndrome and Biventricular Heart Failure in a Patient With Multiple Myeloma

**DOI:** 10.7759/cureus.33538

**Published:** 2023-01-09

**Authors:** Farhan Azad, Ross Moyer, Clive J Miranda, Matthew Gravina

**Affiliations:** 1 Internal Medicine, University at Buffalo, Buffalo, USA; 2 Hematology and Medical Oncology, University at Buffalo, Buffalo, USA

**Keywords:** tumor lysis syndrome, multiple myeloma, proteasome, tls, carfilzomib, bortezomib

## Abstract

Carfilzomib is a proteasome inhibitor (PI) used in multiple myeloma (MM) that is resistant to other therapies. Despite its efficacy and potency, carfilzomib has been associated with kidney injuries, cardiovascular toxic effects, and hematological adverse events. Tumor lysis syndrome (TLS) following the use of PIs in MM, a malignancy not known to cause TLS, has seldom been reported. We present a case of a patient with a known diagnosis of MM who received prior therapy including bortezomib, a first-generation PI, developing worsening heart failure and new onset TLS days after the administration of carfilzomib.

## Introduction

Multiple myeloma (MM) is a clonal expansion of malignant plasma cells in the bone marrow [1]. It is an indolent malignancy, and due to its slow proliferation rate, it is very rare to see tumor lysis syndrome (TLS) in these patients. Reported incidence has been 1% in patients treated with high-dose chemotherapy and approximately 1.4% in patients receiving bortezomib, one of the first proteasome inhibitors (PIs) approved for the treatment of MM [2]. According to product labeling, the frequency of TLS is less than 1% in patients treated with carfilzomib, PI used in our case [3]. Carfilzomib has also been associated with cardiotoxicity, with the extent of toxicity in the literature spanning anywhere from palpitations to cardiac arrest. The most reported manifestation is new-onset or worsening heart failure [4].

MM cells rely on proteasome function to produce secretory proteins necessary for their growth [5]. PIs represent a backbone in current MM therapy and have been shown to improve survival in many clinical trials [6]. The first-in-class bortezomib is a reversible PI that was initially approved for relapsed/refractory MM (RRMM) and later for newly diagnosed MM (NDMM). The second-generation carfilzomib, known for irreversible and more potent proteasome inhibition, was developed to treat RRMM while overcoming the resistance and toxicity profile of bortezomib [7]. We present a case of a patient who received prior bortezomib therapy and developed worsening heart failure and TLS four days after a dose of carfilzomib.

## Case presentation

A 63-year-old African American female with a history of MM diagnosed in 2021, nonischemic cardiomyopathy with an ejection fraction of 45%, diabetes type II, chronic kidney disease (CKD), hypertension, and hyperlipidemia presented from her nursing facility for concerns of shortness of breath and chest pain for three days. She was started on a new chemotherapy regimen, carfilzomib, four days before the presentation. Pertinent home medications included furosemide 20 mg daily, carvedilol 12.5 mg twice a day, irbesartan 75 mg daily, hydralazine 10 mg twice a day, and isosorbide mononitrate 30 mg daily. She denied any symptoms related to dizziness, light-headedness, vision changes, palpitations, orthopnea, or any new changes in her medication regimen and diet. She also denied smoking, drinking alcohol, and using other recreational substances. Family history was insignificant for any malignancy or heart disease.

**Figure 1:**
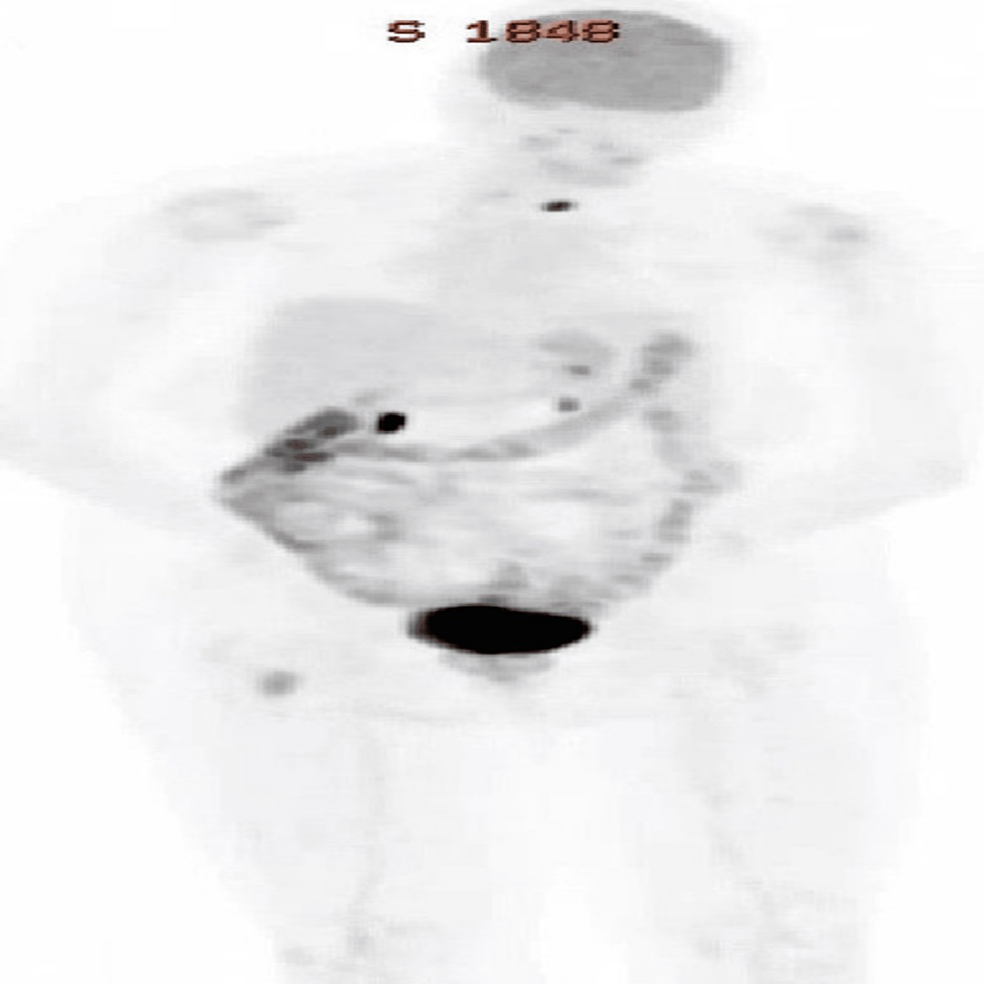
A PET scan showing recurrent metabolically active multiple myeloma involving the right-sided proximal femoral bone. PET, positron emission tomography

Oncological history included MM, IgA kappa type, diagnosed at an outside facility in 2021. The diagnosis was made 18 months before this hospitalization. At the initial visit to the leukemia clinic, she presented with 10% peripheral blood plasma cells, with fluorescence in situ hybridization (FISH) studies revealing *t*(11;14)(q13;q32) immunoglobulin heavy chain/cycle control gene cyclin D1 (IGH/CCND1) rearrangement. Prior therapy included 11 cycles of daratumumab (D), bortezomib (V), lenalidomide (R), dexamethasone (d) (D-VRd) with disease progression and subsequent change in regimen to six cycles of elotuzumab, pomalidomide, and dexamethasone (EloPomDex). Despite therapy, a positron emission tomography (PET) scan done two weeks after the last cycle of EloPomDex revealed recurrent metabolically active MM involving the right-sided proximal femoral bone (Figure 1).

At that time, serum protein electrophoresis (SPEP) showed a large monoclonal protein in the beta fraction, increased from the prior sample. In addition, the patient reported worsening bilateral lower extremity weakness and requiring a walker for ambulation, which was new for her. Due to the patient’s worsening performance status, she was not deemed to be a candidate for high-dose chemotherapy or cellular therapies. Given her progressive disease, treatment was changed to carfilzomib and dexamethasone in addition to venetoclax due to the FISH study findings. It was discussed that if she did not respond to this regimen, she may not have many further treatment options, given her frail status. Four weeks after the last cycle of EloPomDex, she was given cycle one of carfilzomib 36 mg/m^2^, dexamethasone 20 mg, and venetoclax 400 mg. Four days after her therapy, the patient was admitted to the hospital.

On admission, vital signs revealed a heart rate of 140 beats per minute, blood pressure of 121/70 mmHg, respiratory rate of 32 breaths per minute, and temperature of 36.8 °C. Her weight was remarkable at 63.50 kg, down from 72.57 kg four months ago. Oxygen saturation was 82% in room air, and the patient was placed on bilevel-positive air pressure (BiPAP) at a setting of 18/6. After 30 minutes on the BiPAP, her shortness of breath improved but she remained tachypneic. The patient remained alert and oriented, with the ability to protect her airways initially. Bilateral crackles were noted with bilateral pitting edema in the lower extremities. In the emergency department, she was given furosemide 20 mg intravenous injections twice for a presumed heart failure exacerbation. Her laboratory investigations on admission are given in Table 1.

**Table 1:** Initial laboratory investigations. MCV, mean corpuscular volume; PT, prothrombin time; INR, international normalized ratio; PTT, partial thromboplastin time; GFR, glomerular filtration rate; BUN, blood urea nitrogen; BNP, brain natriuretic peptide

Laboratory investigations	Results	Reference range
Complete blood count		
White blood cells	5.6 × 10^9^/L	4.0 × 10^9 ^to 10.5 × 10^9^ L^–1^
Hemoglobin	11.9 g/dL	12-16 g/dL
Hematocrit	36%	37%-47%
MCV	86.3 fL	80-100 fL
Platelet count	166 × 10^9^ L^–1^	140 × 10^9^ to 400 × 10^9^ L^–1^
Coagulation tests		
PT	17.1 seconds	9.4-12.5 seconds
INR	1.40	0.00-3.50
PTT	26.2 seconds	25-35 seconds
Basic metabolic panel		
Sodium level	129 mmol/L	133-147 mmol/L
Potassium level	4.6 mmol/L	3.5-5.6 mmol/L
Chloride	97 mmol/L	96-110 mmol/L
Carbon dioxide	14 mmol/L	20-32 mmol/L
Calcium level	12.5 mg/dL	6.3-11.9 mg/dL
GFR	31 mL/minute/1.73 m^2^	>=60 mL/minute/1.73 m^2^
BUN	58 mg/dL	5-27 mg/dL
Creatinine	1.81 mg/dL	0.40-1.40 mg/dL
Glucose level	244 mg/dL	60-100 mg/dL
Venous blood gas		
pH	7.21	7.35-7.45
CO_2_	51 mmHg	35-45 mmHg
Other laboratory values		
Magnesium	2 mg/dL	1.7-2.7 mg/dL
Phosphate	5.5 mg/dL	2-4.8 mg/dL
Lactate	4.6 mmol/L	0.5-2 mmol/L
BNP	3,258 pg/mL	0-99 pg/mL
Troponin	0.12 ng/mL	0.00-0.06 ng/mL

**Figure 2:**
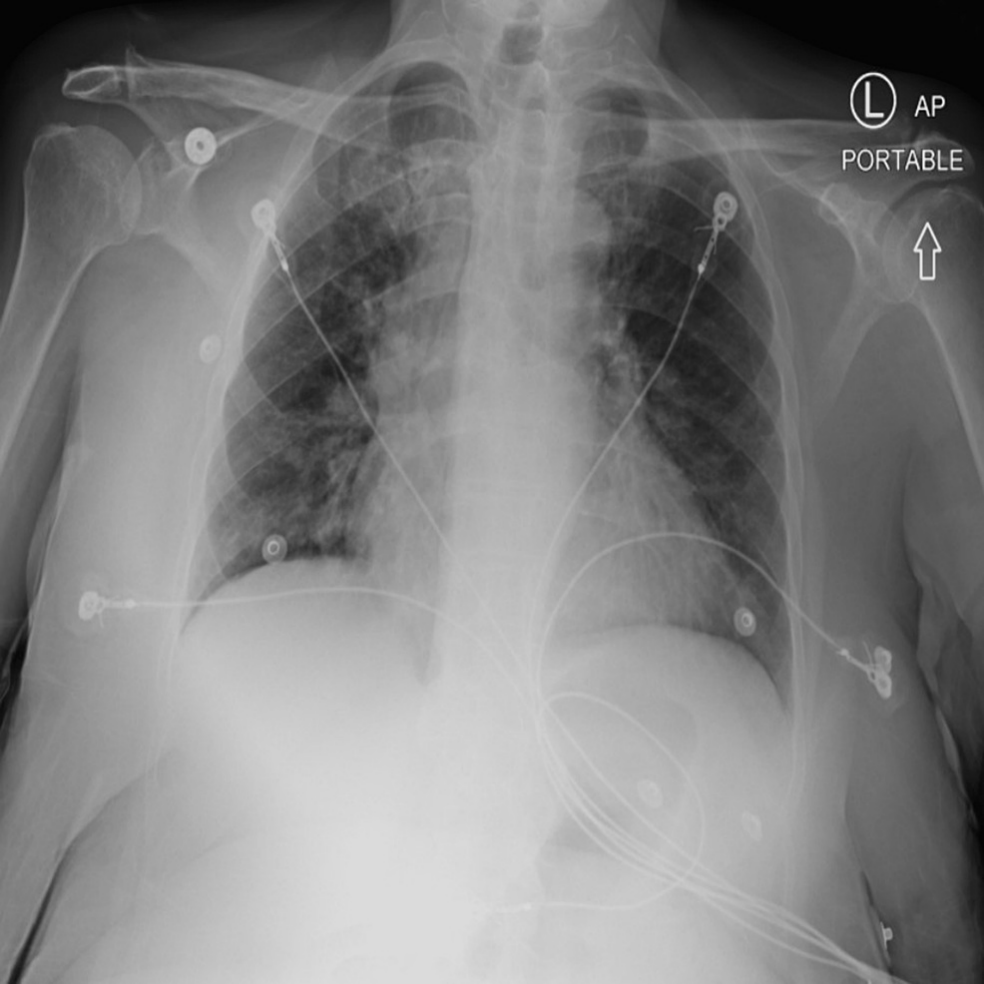
Chest X-ray showing moderately enlarged cardiac silhouette and mild pulmonary vascular congestion.

Electrocardiogram revealed sinus rhythm and a previously noted left bundle branch block, with no ST changes. QTc was noted to be 456 milliseconds. A recent echocardiogram six months ago revealed a left ventricular ejection fraction of 45% and impaired diastolic dysfunction. In addition, it revealed normal sizes of both left- and right-sided cavities. Septal wall motion abnormality was noted, unchanged from a prior echocardiogram 12 months ago. Chest X-ray revealed moderate cardiomegaly and mild pulmonary venous congestion (Figure 2).

Computed tomography angiography of the chest with intravenous contrast was negative for pulmonary embolism but positive for multifocal lung consolidation. Her respiratory status worsened throughout the night, with increased settings on the BiPAP. She started to appear lethargic and confused. Bedside echocardiogram showed a severely reduced ejection fraction of roughly 20% and findings consistent with biventricular failure. No pericardial effusion or tamponade were noted. She was ultimately intubated for airway protection. In addition, the patient became bradycardic and hypotensive requiring pressor support. She tested positive for influenza A, and due to findings in imaging, a mixed cardiogenic and septic shock was suspected. She was started on broad-spectrum antibiotics. Subsequent laboratory investigations in the morning are given in Table 2.

**Table 2:** Subsequent laboratory investigations. MCV, mean corpuscular volume; PT, prothrombin time; INR, international normalized ratio; PTT, partial thromboplastin time; GFR, glomerular filtration rate; BUN, blood urea nitrogen; LDH, lactate dehydrogenase

Laboratory investigations	Results	Reference range
Complete blood count		
White blood cells	7.7 × 10^9^ L^–^^1^	4 × 10^9^ to 10.5 × 10^9^ L^–^^1^
Hemoglobin	10.1 g/dL	12-16 g/dL
Hematocrit	32.4%	37%-47%
MCV	91.3 fL	80-100 fL
Platelet count	136 × 10^9^ L^–^^1^	140 × 10^9^ to 400 × 10^9^ L^–^^1^
Coagulation tests		
PT	21.7 seconds	9.4-12.5 seconds
INR	1.92	0.00-3.50
PTT	36.9 seconds	25.0-35.0 seconds
Basic metabolic panel		
Sodium level	141 mmol/L	133-147 mmol/L
Potassium level	4.9 mmol/L	3.5-5.6 mmol/L
Chloride	97 mmol/L	96-110 mmol/L
Carbon dioxide	14 mmol/L	20-32 mmol/L
Calcium level	7.7 mg/dL	6.3-11.9 mg/dL
GFR	20 mL/minute/1.73 m^2^	>=60 mL/minute/1.73 m^2^
BUN	62 mg/dL	5-27 mg/dL
Creatinine	2.66 mg/dL	0.40-1.40 mg/dL
Glucose level	96 mg/dL	60-100 mg/dL
Venous blood gas		
pH	7.13	7.35-7.45
CO_2_	64 mmHg	35-45 mmHg
Other laboratory values		
Magnesium	2.5 mg/dL	1.7-2.7 mg/dL
Phosphate	10.2 mg/dL	2-4.8 mg/dL
Lactate	15 mmol/L	0.5-2 mmol/L
Troponin	0.91 ng/mL	0.00-0.06 ng/mL
Uric acid	14.8 mg/dL	3-8 mg/dL
LDH	5,258 unit/L	95-265 unit/L
Beta-2 microglobulin	27.64 mg/L	0.80-2.34 mg/L
Fibrinogen	554 mg/dL	200-470 mg/dL
D-dimer	5.35 mcg/mL	0.00-0.45 mcg/mL

Repeat laboratory values were suggestive of TLS. Rasburicase was considered, but given her worsening prognosis and multiorgan dysfunction, it was not given. Her prognosis was discussed with her family, and her code status was changed to reflect *do not resuscitate with comfort measures*. Unfortunately, she expired later in the morning.

## Discussion

TLS is a clinical and biochemical manifestation that can occur spontaneously in highly proliferating tumors or more commonly because of cytotoxic chemotherapy during treatment [8]. It is a hematological emergency diagnosed clinically based on symptoms, context, and laboratory abnormalities. The Cairo Bishop criteria were established to quantify specific laboratory values to aid in diagnosis. It is defined by abnormalities in two of uric acid >8 mg/dL, calcium <7 mg/dL, potassium >6 mEq/L, phosphate >4.5 mg/dL, and one increase in serum creatinine by 1.5 times of upper limit of normal, arrhythmia, and seizures [9]. Our patient rapidly deteriorated, with laboratory values showing both elevated uric acid and phosphate along with increased serum creatinine. In patients treated with carfilzomib, TLS was observed in five patients in phase II trials. To reduce the risk of TLS, patients should be well-hydrated before and after carfilzomib therapy. Euvolemia should be maintained throughout the treatment and monitored to prevent fluid overload, especially in patients with a prior cardiac history [10]. Considering that MM is rarely associated with TLS, prophylactic rasburicase or allopurinol was not considered during the administration of carfilzomib. While our patient did receive intravenous fluids with carfilzomib, she tested positive for the flu, which could have triggered the TLS due to dehydration. While no specific mechanism exists, predisposing factors in these patients include higher baseline uric acid or creatinine, rapidly progressive anemia, and raised lactate dehydrogenase (LDH) and β2-microglobulin, indicating high tumor burden [11]. Laboratory values indicated a high tumor burden in our patient, along with higher baseline creatinine due to prior history of CKD. It might have been useful to consider TLS prophylaxis in our patient during carfilzomib therapy, given her preexisting comorbidities.

It is important to accurately assess cardiovascular risk factors RRMM. The incidence of cardiovascular disease has been described to be higher in MM patients (60.1%) than in non-MM patients (54.5%) [12]. Carfilzomib has been known to cause severe cardiovascular adverse events (CAEs) soon after initiation. It is hypothesized that carfilzomib increases the oxidative stress burden on cardiac myocytes and causes both vascular and endothelial dysfunction due to proteasome inhibition [13-14]. Another proposed mechanism is that carfilzomib may decrease vascular compliance and increase tone [15]. The current rate of incidence is unknown, but one trial showed that up to 50% of patients are affected by CAEs when treated with carfilzomib, the most common event being heart failure. Risk factors for developing these events were those aged over 75 years, obesity, and diagnosis of hypertension [16]. Three randomized trials in RRMM (NCT01080391, NCT01568866, and NCT01302392) showed higher rates of cardiac failure, dyspnea, and hypertension in the carfilzomib arm compared to the control arm [17]. Our patient had a prior history of cardiomyopathy and hypertension, which could have predisposed her to acute onset heart failure with carfilzomib use. Furthermore, a retrospective study by Mangla et al. showed that CAEs due to carfilzomib generally occur early in the treatment course in non-Caucasians [18]. This was seen in our case, as the patient decompensated four days after receiving carfilzomib.

In 2012, the Food and Drug Administration (FDA) approved carfilzomib for MM patients who have received at least two prior therapies, including bortezomib and an immunomodulatory agent, and who have shown disease progression while on therapy or within 60 days of completion of the last therapy [19]. Our patient received both bortezomib and two immunomodulatory agents, lenalidomide and pomalidomide. In the pivotal study that lead to the approval of carfilzomib, PX-171-003-A1, carfilzomib was administered at doses of 20-27 mg/m^2^. In a subsequent clinical trial, the addition of 40 mg of dexamethasone weekly showed an improved overall response rate (ORR) of 55% compared to 50% with carfilzomib alone among patients treated with escalating carfilzomib doses of up to 70 mg/m^2 ^[20]. Given our patient’s cardiac history, the addition of dexamethasone could also have triggered heart failure due to steroid-induced fluid retention. Close monitoring of cardiovascular dysfunction is needed when trialing patients on this new oncologic regimen.

## Conclusions

Our patient developed TLS and worsening heart failure four days after receiving carfilzomib, a potent PI. She had a known diagnosis of multiple myeloma, a malignancy not known to cause TLS. She ultimately had the flu, and subsequent dehydration could have triggered TLS. She also had a prior cardiac history that could have predisposed her to cardiotoxicity associated with carfilzomib. The case outlines the challenge of managing refractory multiple myeloma in a patient with preexisting comorbidities. Patients on carfilzomib therapy should be monitored closely and made aware of the potential risks associated with the medication.
